# Mineral Bioaccessibility and Antioxidant Capacity of Protein Hydrolysates from Salmon (*Salmo salar*) and Mackerel (*Scomber scombrus*) Backbones and Heads

**DOI:** 10.3390/md21050294

**Published:** 2023-05-11

**Authors:** Beatriz de la Fuente, Tone Aspevik, Francisco J. Barba, Katerina Kousoulaki, Houda Berrada

**Affiliations:** 1 Preventive Medicine and Public Health, Food Science, Toxicology and Forensic Medicine Department, Faculty of Pharmacy, Universitat de València, Avda Vicent Andrés Estellés, 46100 València, Spain; beatriz.fuente@uv.es; 2 Department of Nutrition and Feed Technology, Nofima, 5141 Fyllingsdalen, Norway; tone.aspevik@nofima.no (T.A.); katerina.kousoulaki@nofima.no (K.K.)

**Keywords:** fish protein hydrolysates, minerals, bioaccessibility, antioxidant capacity, heavy metals, *Salmo salar*, *Mackerel scombrus*

## Abstract

Information on the bioaccessibility of minerals is essential to consider a food ingredient as a potential mineral fortifier. In this study, the mineral bioaccessibility of protein hydrolysates from salmon (*Salmo salar*) and mackerel (*Scomber scombrus*) backbones and heads was evaluated. For this purpose, the hydrolysates were submitted to simulated gastrointestinal digestion (INFOGEST method), and the mineral content was analyzed before and after the digestive process. Ca, Mg, P, Fe, Zn, and Se were then determined using an inductively coupled plasma spectrometer mass detector (ICP-MS). The highest bioaccessibility of minerals was found in salmon and mackerel head hydrolysates for Fe (≥100%), followed by Se in salmon backbone hydrolysates (95%). The antioxidant capacity of all protein hydrolysate samples, which was measured by Trolox Equivalent Antioxidant Capacity (TEAC), increased (10–46%) after in vitro digestion. The heavy metals As, Hg, Cd, and Pb were determined (ICP-MS) in the raw hydrolysates to confirm the harmlessness of these products. Except for Cd in mackerel hydrolysates, all toxic elements were below the legislation levels for fish commodities. These results suggest the possibility of using protein hydrolysates from salmon and mackerel backbones and heads for food mineral fortification, as well as the need to verify their safety.

## 1. Introduction

Protein hydrolysates produced from fish side stream materials are among the most important added-value products proposed under the circular economy and aquaculture biomass valorization [1,2]. From a nutritional point of view, protein hydrolysates from fish processing side streams are considered to have good-quality protein due to both the ratio of essential and non-essential amino acids and their high digestibility [2]. From a biological function perspective, they are considered to exhibit antioxidant, antimicrobial, anti-hypertensive, antiproliferative, and immunomodulatory activities [3,4,5,6]. Although several fish side streams have been reported to contain relevant amounts of minerals over the last years [7,8,9], these elements are not usually analyzed in fish protein hydrolysates and related products. For instance, Liaset and Espe reported high levels of potassium in protein hydrolysates from salmon and saithe side streams [10]. However, scientific data on this subject are scarce.

Macro minerals such as calcium (Ca), magnesium (Mg), potassium (K), sodium (Na), and phosphorous (P), as well as micro minerals such as iron (Fe), zinc (Zn), copper (Cu), manganese (Mn), cobalt (Co), and selenium (Se) are essential nutrients to maintain normal physiological functions in the human body. While macro minerals are structural and functional components in tissues, micro minerals can act as co-factors in multiple enzymatic reactions [11,12]. Essential minerals must be provided by the diet, and the deficiency of one or more can lead to disorders and diseases [12]. In order to alleviate mineral deficiencies in the population, strategies such as crop biofortification and food fortification have been suggested [12].

The health-related effects of foodstuff constituents depend not only on their initial content but also on their bioavailability. In vivo assays are the point of reference for this purpose, but they are costly and time-consuming and involve ethical concerns. Meanwhile, in vitro digestion assays allow the estimation of bioaccessibility (total soluble food compound released from the food matrix and available for absorption), a prerequisite of bioavailability [13]. The chemical and cellular antioxidant capacities of some protein hydrolysates from different fish side streams submitted to in vitro gastrointestinal digestion have been reported [14,15,16]. However, as far as we know, there are no scientific studies on the bioaccessibility of minerals from fish protein hydrolysates.

In addition to nutritional and bioactive compounds, potential contaminants from both the aquaculture and the marine environment can accumulate in different tissues of farmed and wild fish. The main harmful substances for consumers are mycotoxins derived from plant ingredients in fish diets [17,18], heavy metals from water, particulate matter, and fish-based aquafeeds [19,20], as well as veterinary residues [21]. Therefore, before using fish protein hydrolysates as a means to formulate fortified food products for human consumption, their food safety must be evaluated.

In previous studies, the fish protein hydrolysates analyzed in this work were found to have a well-balanced amino acid composition, reasonable sensory properties, as well an absence of fungal metabolites and mycotoxins [22,23]. The main objectives of this study were to (1) investigate the bioaccessibility of minerals and the antioxidant capacities of the protein hydrolysates and (2) to analyze the content of heavy metals. This will provide useful information about the nutritional potential of fish protein hydrolysates based on salmon backbones (HSB), salmon heads (HSH), mackerel backbones (HMB), and mackerel heads (HMH).

## 2. Results and Discussion

### 2.1. Mineral Content in Fish Protein Hydrolysates

The total content of minerals in the fish protein hydrolysates evaluated in our study is shown in Table 1. In all samples, the predominant macro mineral was P (6832–12,123 µg/g), followed by Mg (822–2773 µg/g) and Ca (789–1786 µg/g). The hydrolysates based on mackerel showed higher levels of all macro minerals compared to those from salmon corresponding to the higher levels of ash present in the mackerel hydrolysates [22]. The HMH sample was especially rich in ash (38.3%), which could explain its high P, Mg, and Ca content [22].

Regarding the micro minerals studied, the concentrations ranged from 11.0 to 15.7 µg/g for Fe, 5.0–74.5 µg/g for Zn, and 0.21–7.77 µg/g for Se (Table 1). Some differences in trace element content were observed between samples. While backbone hydrolysates showed the same decreasing order of trace mineral content (Fe > Zn > Se), it was different for hydrolysates from salmon heads (Zn > Fe > Se) and mackerel heads (Fe > Se > Zn). As expected, due to the presence of gill tissue [24], elevated concentrations of Zn, which were 8- to 15-fold higher than in the rest of the hydrolysates, were found in the HSH sample. In the same way, higher levels of Se were observed in mackerel hydrolysates with respect to salmon hydrolysates. This may be because Se is present in relatively high levels in fish, the natural prey of mackerel, whereas the Se content in salmon tissue depends on the Se levels of the fish meal in their diets, which have declined in recent years [25]. In addition, Se supplementation is strictly regulated by the EFSA (the max added amount that is allowed is a final concentration of 0.5 mg/kg in the diet) [26], while the Se that is present in fish meal can be above 2 mg/kg [27].

Based on a valorization approach, the main macro minerals (Ca, Na, K, Mg, P) and some micro minerals (Fe, Zn, Co, Cu, Mn) have been identified in several side streams of different fish species [8,28,29,30]. According to the data reported, fish side streams containing inorganic structures such as bones are rich in Ca and P, which could contribute to the relevant amounts of these minerals in all fish protein hydrolysate samples. In addition, Zn appears to be the main mineral in fish skin, so the high levels of Zn in HSH could be related to the fish species. For instance, the content of Zn in salmon heads ranged from 51 to 69 µg/g in previous studies [28]. Despite the importance of minerals for human health, there is little information in the literature about the content of minerals in protein hydrolysates based on fish processing side streams. The content of macro and micro minerals in hydrolysates from wild saithe frames, as well as in commercial hydrolysates from unspecified salmon side streams, have been described [10].

### 2.2. Bioaccessibility of Minerals in Fish Protein Hydrolysates

The content of minerals in fish protein hydrolysates before and after gastrointestinal digestion, as well as their bioaccessibility, are reported in Table 2. Before and after the simulated digestion process, the most abundant element was P, followed by Mg and Ca for all hydrolysates. However, there were some differences in the bioaccessibility of these macro minerals. The bioaccessibility values for P were higher in HSB and HMB (13%) than in HSH (10%) and HMH (6%), while the bioaccessibility values for Mg were greater in HSH and HMH (20%) than in HSB (18%) and HMB (14%). These differences could be related to the raw fish side streams used to obtain the hydrolysates (backbones vs. heads), regardless of the type of fish (mackerel vs. salmon). In the case of Ca, the bioaccessibility could only be calculated for the HMH sample (5%). It should be noted that even though HMH showed the highest content of P before the digestion process, its bioaccessibility was the lowest. This highlights the importance of evaluating the effect of the digestion process on nutrients and bioactive compounds in food.

Regarding the micro minerals studied, before digestion, backbone hydrolysates showed the same decreasing order of trace mineral content (Fe > Zn > Se), while it was different for salmon heads (Zn > Fe > Se) and mackerel heads (Fe > Se > Zn) hydrolysates. The best percentages of bioaccessibility were found in Fe from HSH and HMH (≥100%), as well as Se from HSB (95%). A relevant bioaccessibility (50%) of Zn from the HMH sample was also observed. These results could be due to the binding capacity of some minerals to specific amino acids that act as metal ligands to form soluble complexes. For instance, the oyster protein hydrolysate-zinc complex improved Zn bioaccessibility compared to that of various zinc salt solutions [31]. Small peptides from tilapia (*Oreochromis niloticus*) skin collagen also increased zinc-chelating ability and zinc bioaccessibility [32]. Similarly, the iron-binding capacity of protein hydrolysates from mackerel (*Trachurus* japonicas) processing side streams was not significantly affected during in vitro gastrointestinal digestion [33]. Based on the high bioaccessibility obtained in this study, HSH, HMH, and HSB are interesting candidates for Fe, Se, and Zn food fortification since there is a deficit of Fe (60%), Se (30%), and Zn (15%) in the world population, including in both industrial and developing countries [11].

### 2.3. Bioaccessibility of Antioxidant Capacity in Fish Protein Hydrolysates

**Figure 1:**
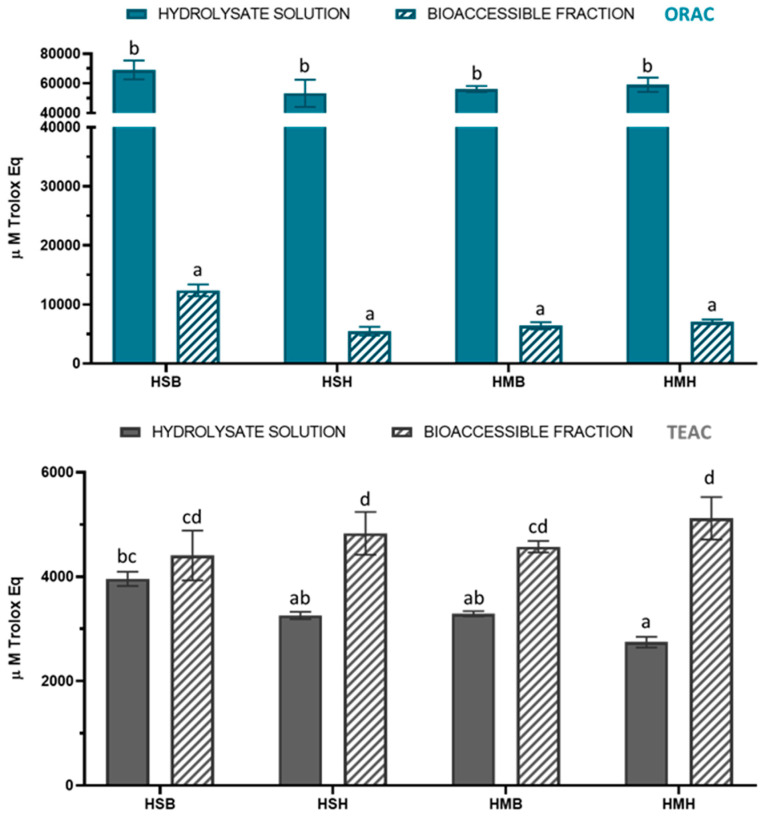
Total antioxidant capacity by TEAC and ORAC before and after simulated gastrointestinal digestion in fish protein hydrolysates. TEAC: Trolox Equivalent Antioxidant Capacity. ORAC: Oxygen Radical Absorbance Capacity. Data are expressed as mean ± SD (n = 3 for TEAC and n = 6 for ORAC). Different lowercase letters in the bars indicate statistically significant differences (*p* < 0.05) among samples.

The total antioxidant capacity of the salmon and mackerel protein hydrolysates was determined by two different approaches, the TEAC and ORAC methods, before and after simulated gastrointestinal digestion (Figure 1). The antiradical activity of the hydrolysates before digestion ranged from 2746 to 3960 and from 53,271 to 69,066 µM Trolox Eq for TEAC and ORAC assays, respectively. The antiradical activity ranges of the hydrolysates after digestion were 4408–5119 µM Trolox Eq (TEAC) and 5488–12,406 µM Trolox Eq (ORAC). Interestingly, the in vitro gastrointestinal digestion produced an increase in TEAC antioxidant capacity as well as a decrease in ORAC antioxidant capacity for all hydrolysate samples. This suggests that the hydrolysates were more efficient in reducing ABTS+ compared with scavenging peroxyl radicals. In particular, TEAC values increased by 10, 28, 32, and 46% for HSB, HMB, HSH, and HMH, respectively. Despite the reduction in ORAC values, the results revealed that the bioaccessible fractions of all fish protein hydrolysates contained antioxidant compounds with different mechanisms of action, which are available to be adsorbed and exert their antioxidant activity. The results are in agreement with other studies that found an increase in antioxidant capacity by the TEAC method after the simulated gastrointestinal digestion of protein hydrolysates from weakfish (*Cynoscion guatucupa*), hake (*Merluccius capensis*), and tilapia (*Oreochromis niloticus*) side streams [14,15,16]. In addition, Zhang et al. [16] showed a positive correlation between the TEAC values of tilapia hydrolysate digesta and cellular antioxidant activity, thus showing the validity of the chemical assay.

The antioxidant properties of fish protein hydrolysates have been related to the presence of low molecular weight peptides and specific amino acids [3,6,34]. During the in vitro digestion, the hydrolysate peptides are broken down into smaller protein fragments by digestive enzymes, which can possess antioxidant properties. However, additional research in this regard is required to correlate specific antioxidant peptides with the antioxidant activity exhibited by salmon and mackerel protein hydrolysates.

### 2.4. Heavy Metal Content in Fish Protein Hydrolysates

The toxic metals As, Hg, Cd, and Pb in fish protein hydrolysates were quantified to ascertain the safety of these products (Table 3). The analyzed heavy metal concentrations were 0.97–1.42 for As, 0.03–0.05 for Hg, 0.01–0.15 for Cd, and 0.08–0.10 for Pb (µg/g). In salmon hydrolysates, the most abundant heavy metal element was As, followed by Pb, Hg, and Cd, while in mackerel hydrolysates, the decreasing order of metal content was As > Cd > Pb > Hg. In agreement with our results, higher levels of As (4–14 µg/g) compared to Hg (<0.03–0.07 µg/g), Cd (<0.01–0.03 µg/g), and Pb (<0.01 µg/g) were observed in protein hydrolysates from salmon and saithe side streams [10]. In addition, Vázquez et al. [35] reported a heavy metal content of 0.48, 0.54, and <0.1 µg/g as well as 35.7, 0.31, and <0.07 µg/g for Cd, Hg, and Pb in protein hydrolysates from monkfish heads and viscera, respectively. Except for Cd in the mackerel hydrolysates, all toxic elements were low and below the limits set by the European Commission for edible fish (<0.05 µg/g) [36].

Following a valorization approach, As, Hg, Cd, and Pb have recently been identified in different side streams of several fish species [8,20,37,38,39,40,41]. Other undesirable compounds, such as PCBs and dioxins, can be found in the fatty tissues of fish, and their levels in the hydrolysates studied here are believed to be low due to the removal of lipids in the hydrolysis process [22]. Our results highlight the need to assess the safety of value-added products based on fish side stream materials. Since fish protein hydrolysates could be used as ingredients in food and pharmaceutical industries, the quantification of potential toxic elements in both the raw material and the final product must be evaluated.

## 3. Materials and Methods

### 3.1. Fish Protein Hydrolysates

Protein hydrolysates based on salmon (*Salmo salar*) and mackerel (*Scomber scombrus*) heads and backbones were provided by the Department of Nutrition and Feed Technology of the Nofima Food, Fisheries and Aquaculture Research Institute (Bergen, Norway). Salmon backbones and heads came from aquaculture, while those of mackerel were from fisheries. The four fish protein hydrolysates used in this work were obtained by enzymatic hydrolysis assisted by the protease FoodPro PNL (EC 3.4.24.28, DuPont, Wilmington, DE, USA) and under mild conditions (50 °C) for 60 min [22].

### 3.2. Reagents

#### 3.2.1. In Vitro Gastrointestinal Digestion

Pepsin (porcine, 975 units per mg protein), pancreatin (porcine, activity equivalent to 8× USP specifications), and bile extract (porcine) were supplied by Sigma (St. Louis, MO, USA). Ammonium carbonate ((NH_4_)_2_CO_3_), calcium chloride dihydrate (CaCl_2_(H_2_O)_2_), magnesium chloride hexahydrate (MgCl_2_(H_2_O)_6_), potassium chloride (KCl), sodium chloride (NaCl), potassium dihydrogen phosphate (KH_2_PO_4_), and sodium bicarbonate (NaHCO_3_) were provided from Merck (Darmstadt, Germany). Culture-grade water was purchased from B. Braun (Melsungen AG, Germany).

#### 3.2.2. Essential and Heavy Metals

Single-component standards of Mg, Ca, P, Fe, Zn, and Se (1 µg/mL) and Ar, Hg, Cd, and Pb (1 g/L) were obtained from High-Purity Standards (North Charleston, South Caroline), while internal isotope standard solutions of Sc, Ge, Rh, and Ir (20 µg/g) were purchased from ISC Science (Gijón, Spain). Nitric acid (69%) for ppb-trace grade analysis and hydrogen peroxide (35%) for analysis were purchased from Scharlab (Barcelona, Spain).

#### 3.2.3. Antioxidant Capacity

ABTS (2,20-azinobis (3-ethylbenzothiazoline 6-sulfonic acid)), AAPH (2,20-azobis (2-amidinopropane), Trolox^®^ (6-hydroxy-2,5,7,8-tetramethylchroman-2-carboxylic acid), sodium fluorescein, and potassium persulfate (K_2_S_2_O_8_) were purchased by Sigma (St. Louis, MO, USA). Potassium phosphate monobasic (Na_2_HPO_4_), potassium phosphate dibasic (K_2_HPO_4_), and ethanol (96%) were obtained from Merk (Darmstadt, Germany). Water was deionized using a Milli-Q SP^®^ ReagentWater system (resistivity > 18 MW cm^−1^, Millipore Corporation, Bedford, MA, USA).

### 3.3. Simulated Gastrointestinal Digestion Process

The fish side stream protein hydrolysates were diluted in culture-grade water (1/10 *m*/*v*) to reach at a final concentration of 4% of proteins according to Egger et al. [42]. The standardized method INFOGEST was applied [43]. For this, simulated gastrointestinal fluids were prepared, and enzyme activity assays were performed. Orbital shaking in a water bath (Stuart SBS30, Staffordshire, UK) was used to simulate the gastrointestinal digestion process. Because of the lack of carbohydrates in the hydrolysate samples, the salivary step was carried out without amylase enzyme.

Briefly, 2.5 mL of hydrolysate solution, 2 mL of salivary fluid, 12.5 μL of 0.3 M CaCl_2_, and culture-grade water to a final volume of 5 mL were mixed for 2 min. Afterwards, to simulate the gastric phase, 3.75 mL of gastric fluid, 0.8 mL of pepsin solution (25,000 U/mL), and 2.5 μL of 0.3 M CaCl_2_ were added. Then, the pH was adjusted at 3.0, culture-grade water was added to a final volume of 10 mL, and the gastric mixture was then incubated for 2 h. Subsequently, 5.5 mL of intestinal fluid containing pancreatin (100 U trypsin activity/mL) and bile salts (10 mmol/L) and 20 μL of 0.3 M CaCl_2_ were added. Next, the pH was adjusted to 7.0, culture-grade water was added up to a final volume of 20 mL, and the intestinal mixture was then incubated for 2 more hours. The gastrointestinal steps were simulated under mechanical shaking at 95 rpm and 37 °C. After in vitro digestive process, samples were cooled (ice bath) and centrifuged (3100 g/4 °C/30 min). The obtained supernatant solution corresponded to the bioaccessible fraction. Blank 2.5 mL samples of culture-grade water were subjected to the same simulated gastrointestinal digestion, and their bioaccessible fraction values were subtracted from the values of the digested hydrolysate samples. The bioaccessibility was calculated as the ratio between the content in the bioaccessible fraction and the initial content in the hydrolysate solution. The results were expressed as percentage of bioaccessibility according to the following equation:

Bioaccessibility (%) = (content in bioaccessible fraction/initial content) × 100.

### 3.4. Analysis of Minerals

The content of macro minerals (Mg, Ca, P) and micro minerals (Zn, Fe, Se) in the protein hydrolysates and their corresponding bioaccessible fractions was evaluated. Approximately 10 mg of solid samples and 1 mL of liquid samples were placed in a Teflon reactor vessel. Afterwards, 1 mL of HNO_3_ and 250 µL of H_2_O_2_ were added. The acid mineralization was conducted using high-pressure microwave digester irradiation (Ethos Easy, Milestone, Sorisole, Italy) at 800 W and 180 °C for 15 min. After treatment, the samples were left to cool at room temperature and filtered. Hydrolysates and bioaccessible fractions were made up to a final volume of 10 and 5 mL with distilled water, respectively. All solutions were diluted 1/100 before analysis.

The identification and quantification of minerals was performed using an inductively coupled plasma spectrometer mass detector (ICP-MS, Agilent model 7900). The operating conditions were as follows: carrier gas (1.0 L/min), Ar gas flow (15.0 L/min), RF power (1550 W), nebulizer pump speed (0.30 rps), and RF matching (1.80 V). Different standard calibration curves were used for the quantification of minerals: 10–8000 ppb for Ca and Mg; 5–2000 ppb for Fe and Zn; 5–1000 ppb for Se; and 10–4000 for S. Limits of detection (LOD) were calculated according to the following equation: LOD = 3sB/a, where 3sB is 3 times the standard deviation at zero concentration and a is the slope of the calibration curve. LOD values were 5 ppb for Ca and S, 1 ppb for Fe and Se, 0.5 ppb for Mg, and 0.05 ppb for Zn. Distilled water was used as a blank. Depending on the sample, the results were expressed as µg of mineral/g of hydrolysate or µg of mineral/mL of BF. Dried olive leaves (GSC-FOL/2018) used in intercomparative trials were provided by the Spectroscopy Service of the University of Valencia and considered as a control sample for mineral content. It was analysed in parallel to hydrolysate samples and blank in order to confirm the accuracy of the method. The recovery percentages were 106% (Mg), 92% (Ca), 104% (P), 104% (Zn), and 101% (Fe).

### 3.5. Determination of Antioxidant Capacity

The total antioxidant capacity in the fish hydrolysate solutions prepared for the gastrointestinal simulation and in their corresponding bioaccessible fractions was performed by two methods with different mechanisms of antioxidant action, Trolox Equivalent Antioxidant Capacity (TEAC) and Oxygen Radical Absorbance Capacity (ORAC). Both antioxidant assays were applied in a previous work for protein extracts from salmon side streams [39].

### 3.6. Analysis of Heavy Metals

The content of As, Hg, Cd, and Pb in the fish protein hydrolysates was evaluated. Approximately 0.2 g of sample, 1 mL of H_2_O_2_ (30% *v*/*v*), and 4 mL of HNO_3_ (14 M) were placed in a Teflon reactor vessel. Then, the acid digestion was carried out by microwave irradiation at 800 W and 180 °C for 15 min (MARS, CEM, Vertex, Barcelona, Spain). The digested hydrolysates were left to cool at room temperature, filtered and made up to volume with distilled water.

The identification and quantification of heavy metals were conducted by an inductively coupled plasma spectrometer mass detector (ICP-MS, Agilent model 7900). The analytical conditions were as follows: carrier gas (1.07 L/min), Ar gas flow (15.0 L/min), reaction gas (He), RF power (1550 W), nebulizer pump speed (0.10 rps), and RF matching (1.80 V). Standard calibration curves from 0 to 1000 µg/L were used for the quantification of As, Cd, and Pb. A standard calibration curve from 0 to 100 µg/L was used for Hg. Limits of detection (LOD) were calculated according to the following equation: LOD = 3sB/a where, 3sB is 3 times the standard deviation at zero concentration and a is the slope of the calibration curve. LOD values were 0.0015 µg/L for Hg and Pb, 0.012 µg/L for As, and 0.004 µg/L for Cd. Distilled water was used as a blank. The results were expressed as µg of heavy metal/g of hydrolysate. The Certified Reference Material for Trace Metals DORM-3 (fish protein powder) was used to confirm the accuracy of the method, which was analyzed at the same time as the samples and blank. The recovery percentages were 98%, 86%, 76%, and 77% for As, Hg, Cd, and Pb, respectively.

### 3.7. Statistical Analysis

Significance differences among samples were analyzed by one-way analysis of variance (ANOVA) and Tukey Honestly Significant Difference (HSD) multiple range test (*p* < 0.05) using the Statgraphics Centurion XVI^®^ software (Statpoint Technologies, Inc., Warrenton, VA, USA).

## 4. Conclusions

Simulated gastrointestinal digestion affected the bioaccessibility of both the minerals and the antioxidant capacity of protein hydrolysates from salmon and mackerel backbones and heads. In particular, Fe and Se showed high bioaccessibility from protein hydrolysates based on heads. After the in vitro digestion process, the antioxidant capacity measured by TEAC increased, thus confirming a potential antioxidant effect to be exerted in the body. Bioavailability assays using cell cultures could be applied to assess the mineral uptake from the hydrolysates as well as the antioxidant properties of the absorbed fraction.

Except for Cd in mackerel hydrolysates, all toxic metals were below the legislation levels for fish products. Overall, this work represents complementary information for the future utilization of protein hydrolysates from farmed salmon and wild mackerel backbones and heads.

## Figures and Tables

**Table 1:** Concentrations of minerals in fish protein hydrolysates.

Minerals (µg/g)						
Hydrolysate	Mg	Ca	P	Fe	Zn	Se
HSB	1042 ± 15	789 ± 18	8370 ± 50	11.0 ± 0.2	7.1 ± 0.2	0.21 ± 0.01
HSH	822 ± 10	789 ± 20	6832 ± 400	12.4 ± 0.2	74.5 ± 0.8	0.98 ± 0.03
HMB	2326 ± 50	935 ± 30	11,569 ± 600	15.7 ± 0.4	9.7 ± 0.5	4.89 ± 0.01
HMH	2773 ± 30	1786 ± 30	12,123 ± 700	14.4 ± 0.5	5.0 ± 0.3	7.77 ± 0.01

Data are expressed as the mean ± SD. The SD values refer to the variability of the technique (four measurements of each sample).

**Table 2:** Mineral content before and after simulated gastrointestinal digestion in fish protein hydrolysates.

Hydrolysate	Hydrolysate Solution	Bioaccessible Fraction	Bioaccessibility
Magnesium (mg/L)			
HSB	104 ± 1	18.9 ± 0.2	18%
HSH	82 ± 1	16.6 ± 0.4	20%
HMB	233 ± 6	33.7 ± 0.9	14%
HMH	277 ± 3	56.2 ± 0.9	20%
Calcium (mg/L)			
HSB	79 ± 2	<5	nd
HSH	79 ± 2	<5	nd
HMB	94 ± 3	<5	nd
HMH	179 ± 3	8.4 ± 0.8	5%
Phosphorus (mg/L)			
HSB	837 ± 5	106 ± 9	13%
HSH	683 ± 40	68 ± 11	10%
HMB	1157 ± 60	145 ± 11	13%
HMH	1212 ± 70	74 ± 8	6%
Iron (mg/L)			
HSB	1.10 ± 0.02	0.21 ± 0.01	19%
HSH	1.24 ± 0.02	1.33 ± 0.05	112%
HMB	1.57 ± 0.04	0.21 ± 0.02	13%
HMH	1.44 ± 0.05	1.52 ± 0.06	105%
Zinc (mg/mL)			
HSB	0.71 ± 0.02	0.27 ± 0.04	36%
HSH	7.4 ± 0.08	0.87 ± 0.03	12%
HMB	0.97 ± 0.05	0.33 ± 0.05	34%
HMH	0.50 ± 0.03	0.25 ± 0.04	50%
Selenium (µg/L)			
HSB	21 ± 1	20 ± 2	95%
HSH	98 ± 3	11 ± 3	11%
HMB	489 ± 1	53 ± 5	13%
HMH	774 ± 1	171 ± 6	11%

Data are expressed as the mean ± SD. The SD values refer to the variability of the technique (four measurements of each sample). nd: not detected.

**Table 3:** Concentrations of toxic elements in fish protein hydrolysates.

Minerals (µg/g)				
Hydrolysate	As	Hg	Cd	Pb
HSB	1.107 ± 0.006	0.047 ± 0.001	0.015 ± 0.001	0.083 ± 0.002
HSH	1.421 ± 0.012	0.029 ± 0.001	0.006 ± 0.001	0.093 ± 0.001
HMB	1.379 ± 0.015	0.044 ± 0.005	0.124 ± 0.009	0.048 ± 0.001
HMH	0.969 ± 0.016	0.044 ± 0.002	0.150 ± 0.003	0.104 ± 0.002
Legislation *	<13.5	<0.50	<0.05	<0.30

Data are expressed as the mean ± SD. The SD values refer to the variability of the technique (four measurements of each sample). * Legislation: Values referred to fish muscle tissue according to the European Commission (EC No 1881/2006) [36].

## Data Availability

Not applicable.
